# A Multi-View Deep Learning Model for Thyroid Nodules Detection and Characterization in Ultrasound Imaging

**DOI:** 10.3390/bioengineering11070648

**Published:** 2024-06-25

**Authors:** Sanaz Vahdati, Bardia Khosravi, Kathryn A. Robinson, Pouria Rouzrokh, Mana Moassefi, Zeynettin Akkus, Bradley J. Erickson

**Affiliations:** 1 Artificial Intelligence Laboratory, Department of Radiology, Mayo Clinic, 200 1st Street, SW, Rochester, MN 55905, USA; 2 Department of Radiology, Mayo Clinic, 200 1st Street, SW, Rochester, MN 55905, USA; 3 Department of Laboratory Medicine and Pathology, Mayo Clinic, Jacksonville, FL 32224, USA

**Keywords:** deep learning, ultrasound, thyroid nodule assessment

## Abstract

Thyroid Ultrasound (US) is the primary method to evaluate thyroid nodules. Deep learning (DL) has been playing a significant role in evaluating thyroid cancer. We propose a DL-based pipeline to detect and classify thyroid nodules into benign or malignant groups relying on two views of US imaging. Transverse and longitudinal US images of thyroid nodules from 983 patients were collected retrospectively. Eighty-one cases were held out as a testing set, and the rest of the data were used in five-fold cross-validation (CV). Two You Look Only Once (YOLO) v5 models were trained to detect nodules and classify them. For each view, five models were developed during the CV, which was ensembled by using non-max suppression (NMS) to boost their collective generalizability. An extreme gradient boosting (XGBoost) model was trained on the outputs of the ensembled models for both views to yield a final prediction of malignancy for each nodule. The test set was evaluated by an expert radiologist using the American College of Radiology Thyroid Imaging Reporting and Data System (ACR-TIRADS). The ensemble models for each view achieved a mAP0.5 of 0.797 (transverse) and 0.716 (longitudinal). The whole pipeline reached an AUROC of 0.84 (CI 95%: 0.75–0.91) with sensitivity and specificity of 84% and 63%, respectively, while the ACR-TIRADS evaluation of the same set had a sensitivity of 76% and specificity of 34% (*p*-value = 0.003). Our proposed work demonstrated the potential possibility of a deep learning model to achieve diagnostic performance for thyroid nodule evaluation.

## 1. Introduction

Thyroid cancer accounts for the most common endocrine malignancy. It has an incidence rate of 14.6 per 100,000 people in the United States and is the seventh most common cancer among American women [1,2]. Ultrasound imaging is the primary method to evaluate thyroid nodules. It offers detailed images that assist in evaluating the nodules’ dimensions, form, and composition. Ultrasound modality can help determine whether a nodule needs further evaluation with Fine Needle Aspiration (FNA). While ultrasound is a safe and inexpensive method, FNA is an invasive and costly procedure that confirms the diagnosis of thyroid nodules [3,4]. The majority of thyroid nodules assessed by ultrasound imaging are benign and need no further invasive procedures. Ultrasound imaging interpretation is highly dependent on the reader’s experience, and many thyroid nodules are unnecessarily biopsied. These cases have a high rate of benign nodules in addition to nondiagnostic cases [5]. The American College of Radiology introduced the Thyroid Imaging Reporting and Data System (ACR-TIRAD) to standardize thyroid nodule assessment based on size and features [6]. Although the ACR-TIRAD scoring system has enhanced the inter-reader agreement [7], it has been reported that this scoring system has led to moderate inter-reader agreement among radiologists for thyroid nodule classification. This level of agreement is still lower than clinically desired and maintains the challenge of accurate thyroid nodule characterization [8,9]. The subjective nature of ultrasound interpretation and the high number of unnecessary biopsies highlight the need for improved diagnostic tools in thyroid nodule assessment [10]. In recent years, there has been a proliferation of computer-aided systems designed to assist radiologists in the rapid and accurate diagnosis of thyroid nodules, aiming to mitigate subjective error rates. Artificial intelligence (AI) and machine learning have provided valuable insights and decision support in analyzing medical images in radiology settings [11]. By leveraging advanced algorithms and machine learning techniques, these systems can assist in the interpretation of ultrasound imaging, provide valuable insights for clinical decision-making, and help improve the quality of clinical workflow [12]. In recent years, deep learning, a branch of machine learning, has played a significant role in evaluating different types of malignancies. Likewise, there has been a surge in research focused on harnessing the power of deep learning models for the assessment of thyroid nodule malignancy [13]. Many studies have demonstrated the capability of deep learning algorithms for thyroid nodule evaluation and investigated their potential to assist radiologists in clinical settings for optimal patient care [14]. Several studies focused on thyroid nodule detection and classification in addition to feature extraction from nodules and nodule segmentation [15]. Deep learning architectures have been introduced for the characterization of thyroid nodules and highlight the effectiveness of these algorithms in distinguishing between malignant and benign thyroid nodules. Alghanimi et al. [16] proposed a CNN and ResNet50 model design for the classification of thyroid nodules. In another work, Song et al. [17] utilized InceptionV3 for their model development and demonstrated that their model’s diagnostic performance is comparable with radiologists. Zhu et al. [18] conducted a meta-analysis on the application of VGGNet for thyroid nodule characterization and reported that the deep learning VGGNet model has a reliable and effective performance in the classification of thyroid nodules into benign and malignant. Namsena et al. [19] built their model upon Densenet121 and WSDAN and indicated that their model achieved superior performance in terms of sensitivity and comparable specificity to experienced radiologists. In another study by Snakar et al., XGBoost achieved higher accuracy in comparison to other conventional machine learning algorithms for thyroid disease prediction [20]. Furthermore, the ensembling technique has been introduced as a robust approach utilized in medical imaging classification tasks [21]. Sherma et al. [22] proposed that the ensemble model achieves a better performance in terms of detecting thyroid nodule malignancy compared to base models. As highlighted, research efforts have shown that deep learning models can achieve diagnostic performance comparable to, or even surpass, that of experienced radiologists [23]. These advancements underscore the transformative impact of deep learning on the field of thyroid nodule evaluation, enabling more precise and effective clinical decision-making. On the other hand, studies have reported that transverse and longitudinal views of thyroid nodules contain independent predictors for thyroid nodule malignancy [24]; however, most studies on thyroid ultrasound using deep learning are based on a single image in one plane [25]. Building upon this foundation, our study proposes a novel deep learning-based pipeline designed to detect thyroid nodules in B-mode ultrasound images. The pipeline utilizes two transverse and longitudinal imaging views using an ensembling technique and applies the XGboost model to classify thyroid nodules into benign or malignant groups. In addition to evaluating the performance of our proposed deep learning model in classifying thyroid nodules, we compare its results with a radiologist’s interpretation using the widely adopted ACR-TIRAD scoring system. By conducting a comprehensive comparative analysis, we aim to determine the efficacy and potential advantages of our deep learning-based approach in thyroid nodule classification.

## 2. Methods

**Figure 1:**
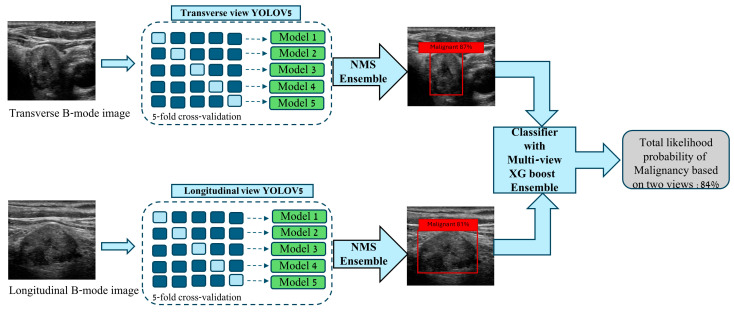
Diagram of a multi-view deep learning architecture with two ensembles, YOLOV5 and XGBoost, for detection and characterization of a thyroid nodule.

This retrospective study was approved by the institutional review board. We randomly collected the B-mode ultrasound images of 983 patients with transverse and longitudinal views. We included patients with solitary thyroid nodules who underwent FNA with pathologic confirmation at Mayo Clinic, Rochester, Minnesota. The patients underwent FNA based on suspicion of thyroid nodule malignancy by being evaluated by a radiologist. Our dataset consists of various types of thyroid nodules, including papillary carcinoma, follicular adenoma, follicular carcinoma, Hurthle cell adenoma, and Hurthle cell carcinoma. We did not include patients who had indeterminate FNA results. For training purposes, we split our dataset at the patient level into training, validation, and test sets. We used the Python package sci-kit-learn to split our dataset [26]. A total of 81 cases were held out as a testing set, and 902 cases were used for training and validation purposes. Our deep learning pipeline consists of three main steps: (1) Detecting the thyroid nodule in ultrasound images. (2) Classifying the nodule into benign or malignant from transverse and longitudinal views separately. (3) Finally, ensembling the predictions from both views to conclude the predicted diagnosis. We converted our images to PNG format during data processing, padded and resized them to 256 × 256 pixels as needed. All nodules were segmented, and the region of interest (ROI) was collected for all images. We utilized each nodule’s ROI to obtain bounding boxes for object detection training purposes. This conversion ensured that bounding boxes accurately represented the relevant areas of the nodules within each image. To detect and characterize thyroid nodules, we trained two separate You Look Only Once (YOLO) version 5 models, one for transverse and one for longitudinal views. YOLO is a robust object detection model widely utilized across various medical imaging applications [27]. YOLOv5 architecture comprises three core sections: backbone, neck, and output. The backbone, which is built upon the Cross Stage Partial Network (CSPNet) and spatial pyramid pooling (SPP) architectures, is a convolutional neural network that extracts essential features from input images and subsequently constructs a feature map. The neck component leverages Path Aggregation Network (PANet) and Feature Pyramid Network (FPN) architectures to aggregate feature maps from the backbone step and create a feature pyramid. The feature pyramid enables the model to detect similar types of objects in varying sizes. The output generates the final output vectors with bounding box coordinates for the detected objects and class probabilities [28]. We leveraged the YOLOv5 model for our pipeline due to its balance between high accuracy and inference speed, which is essential for practical clinical applications that require swift and reliable results. YOLOv5’s state-of-the-art performance in object detection tasks, combined with its efficiency when working with smaller datasets, rendered it an ideal choice, given our retrospective study’s data constraints. Furthermore, its architectural design is conducive to ensemble learning techniques, thereby enhancing the robustness of our predictions across both transverse and longitudinal views. The broad community support and detailed documentation available for YOLOv5 also facilitated seamless implementation and optimization, ensuring the reliability of our models throughout the study. In the current pipeline, YOLOV5 models were tasked to localize each nodule within the image and predict their malignancy probability. Five-fold cross-validation was used to have a more robust set of hyperparameters. We used the popular Scikit learn python library and partitioned our training dataset at the patient level into 5 disjoint sets [26]. For each view, we utilized these five models and ensembled them using non-max suppression (NMS) to boost their collective generalizability. This approach helps to refine the results by eliminating redundant predictions and ensuring that the most relevant predictions are retained. [21] Following this step, we trained an extreme gradient boosting machine (XGBoost) model on the outputs of models for both transverse and longitudinal views to yield a final prediction of malignancy for a single nodule (Figure 1). XGBoost is a comprehensive tree-boosting system. The fundamental concept of gradient boosting involves constructing a sequence of decision trees, where each new tree is designed to rectify the errors made by the preceding trees [29]. One of it’s advantages is that it can incorporate Lasso and Ridge regularization techniques in order to prevent overfitting and enhance the generalizability of the model, ensuring to attain a reliable prediction. XGBoost has been demonstrated to be highly potent in thyroid disease classification tasks. [30]. Mean average precision at 50% overlap (mAP0.5) was used to evaluate the YOLOv5 models, which shows the performance of the model in terms of detecting thyroid nodules in the image, bounding box placement, and class prediction. The whole pipeline’s performance was evaluated using the Area Under the Receiver Operating Curve (AUROC). The ROC curve illustrates the performance of our model across various threshold settings, and the AUC provides a single scalar value to summarize this performance. All the metrics were reported for the holdout test set. The 95% confidence interval (CI) was calculated based on bootstrapping with 1000 iterations.

In our study, we applied various augmentation techniques to enhance the diversity and generalization of our dataset. Random rotations within a range of 50 degrees introduced slight variations in object orientation, improving viewpoint robustness. We applied a translation factor of 0.1 to shift images horizontally and vertically, simulating object displacements and handling location variations. Images were randomly scaled by a factor of 0.9, mimicking objects appearing at different sizes in real-world scenarios. With a probability of 0.5, we performed vertical and horizontal flips to handle reflections and capture object features from different viewpoints. We calculated the cross-sectional nodule area to evaluate the impact of nodule size on our model prediction performance. Employing the mosaic technique, we combined multiple images into a complex mosaic, diversifying the input and enhancing the object detection task. With a probability of 0.5, mixup augmentation blended pairs of images and labels, generating training samples between originals, promoting better generalization and reducing overfitting. For all experiments, the weights were transferred from an ImageNet pre-trained model available in the YOLOV5 repository. It is demonstrated that transfer learning-based models improve the overall performance of classification algorithms, including thyroid nodule classification [31]. During inference, we used a confidence threshold of 0.001 and an intersection over union (IOU) of 0.6. We established our experiment using Python version 3.9, PyTorch framework, and GPU A100 (NVIDIA, Santa Clara, CA, USA), with a batch size of 32 and 100 epochs.

To enhance the evaluation of our model’s performance, a radiologist (K.A.R.) with over ten years of ultrasound imaging experience assessed images in our test set using the ACR-TIRAD scoring system. The radiologist’s evaluation of the thyroid nodules was conducted without the knowledge of the model’s predictions or the patients’ final pathological diagnoses. To compare the predictions of our deep learning model with the ACR-TIRAD scores reported by the radiologist, we established a threshold of 3 as the cut-off point for suggesting nodules as suspicious of malignancy. The model’s classification of thyroid nodules into benign or malignant categories was compared with ACR-TIRAD ≤ 3 and ACR-TIRAD > 3. To assess the significance of the difference between our model’s predictions and the ACR-TIRAD scores, we employed a statistical analysis using the Chi-squared test. By conducting the Chi-squared test, we aimed to evaluate whether there was a statistically significant difference between the classifications made by our model and the classifications based on the ACR-TIRAD scoring system. The significance level was set at 0.05 in all instances.

## 3. Results

**Figure 2:**
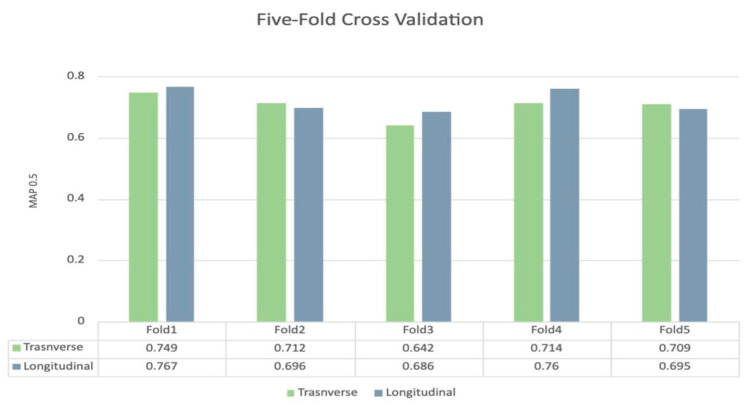
Five-fold cross-validation for transverse and longitudinal YOLOV5 model.

The total number of malignant nodules was 506 (of 983 nodules, 51%); in the test set, 38/81 (46%) of nodules were malignant. On the training set, the mean Average Precision at 0.5 Intersection over Union (mAP0.5) for the five cross-validation folds was 0.70 (Standard Deviation [SD]: 0.038) for the transverse YOLOv5 models and 0.72 (SD: 0.039) for the longitudinal YOLOv5 models. The mAP 0.5 for each fold in each view is demonstrated in Figure 2.

**Figure 3:**
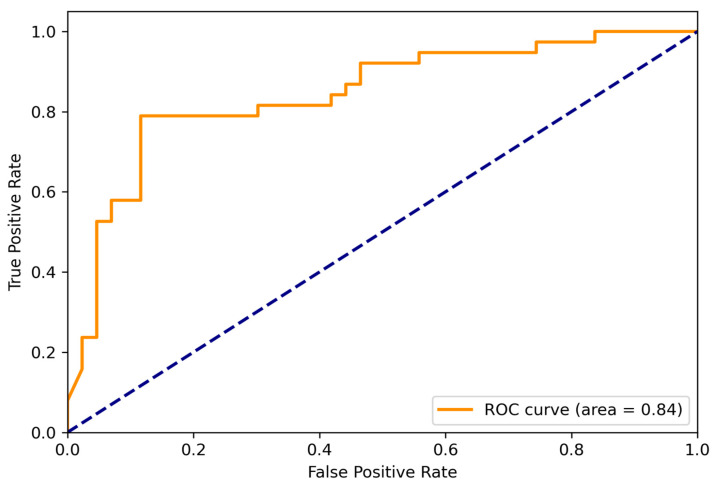
Receiver Operating Curve (ROC) of the ensembled model. AUC = Area Under ROC curve.

The ensemble models for each view achieved a mAP0.5 of 0.797 for the transverse view and 0.716 for the longitudinal view. The entire pipeline, constructed using the XGBoost ensemble, reached an AUROC of 0.84 (95% CI: 0.75–0.91). It achieved 84% sensitivity and 63%specificity in classifying benign versus malignant thyroid nodules (Figure 3). Our model obtained a negative predictive value of 80%, with an F1 score of 0.76. We calculated the cross-sectional nodule area and compared the model’s prediction performance on the lower and higher quartiles of nodule size. We found that the model had approximately similar performances, AUC = 0.81 and AUC = 0.82 for lower and higher quartiles, respectively. This analysis underscores the robustness of our deep-learning model in assessing thyroid nodules of varying sizes.

The radiologist’s ACR-TIRAD scores were collected for the test set. An ACR-TIRAD score of 3 was set as the cut-off value for the evaluation of the diagnostic performance of an expert radiologist. The ACR-TIRAD ≤ 3 was considered as nodules with a higher chance of being benign versus ACR-TIRAD > 3 as nodules suspicious of malignancy. The sensitivity and specificity of the ACR-TIRAD were 78% and 34%, respectively, which was comparably lower than the model prediction (*p*-value = 0.003). We conducted a sensitivity analysis for ACR-TIRAD scoring, which is presented in Table 1 and Table 2 details a comparison between our model’s performance, the ground truth, and the ACR-TIRAD score as evaluated by an expert radiologist. As demonstrated in Table 2, our model did not predict any benign nodule with TIRAD ≤ 3 as malignant; thus, in patients with ACR-TIRAD ≤ 3, the model had a specificity of 100% (15/15) and a negative predictive value of 83% (15/18). On the other hand, in patients with ACR-TIRAD > 3, our model achieved a specificity of 82% (23/28), with a negative predictive value of 84% (27/32).

## 4. Discussion

Thyroid nodules represent a prevalent form of endocrine malignancy, and ultrasound imaging serves as the primary screening method for their detection and assessment. However, the evaluation of thyroid nodule images poses considerable challenges due to the inherent complexity and variability in their appearance, making interpretation of the thyroid nodules highly subjective and reliant on the experience and expertise of radiologists [32]. Over the past few years, there has been a substantial increase in the number of studies focused on harnessing the capabilities of AI models for the assessment of thyroid nodule malignancy [33]. In the current study, we demonstrate that an ensemble of deep-learning models receiving transverse and longitudinal views of thyroid ultrasound B-mode images is more robust in detecting and characterizing thyroid nodules. By integrating the outputs of the ensembled YOLO models into an XGBoost model, we developed a sophisticated pipeline that combines deep learning for feature extraction with classical machine learning for final classification. Our study is among a few studies that integrate both transverse and longitudinal views of thyroid ultrasound images to enhance the accuracy of nodule classification. This dual-view approach leverages complementary information from different perspectives, which is not typically utilized in existing single-view methods. In addition, we compared the performance of our model with the ACR-TIRAD scores reported by an expert radiologist to demonstrate its potential capability for further engagement in clinical settings.

Studies have examined the application of deep learning in classifying thyroid nodules, paving the way for advancements in this field. Ma et al. [34] conducted the first attempt to employ convolutional neural networks for thyroid nodule classification and built their model based on the fusion of two convolutional neural networks. Since then, many deep-learning applications have been introduced for thyroid nodule assessment. Wang et al. [35] established a deep-learning model with the integration of YOLOv2 and Resnet v2–50 for thyroid nodule recognition and classification. One of the limitations of their study was that the malignant nodules in their dataset mainly consisted of thyroid papillary carcinoma. Likewise, He et al. [36] prospectively evaluated an AI model’s performance compared to senior and junior radiologists, and they reported that they solely used papillary thyroid carcinoma for their experiments. In a recent review of deep learning applications for thyroid nodule analysis, Slabaugh et al. [37] demonstrated that papillary thyroid carcinoma is particularly investigated among thyroid lesions for imaging analysis. However, the clinical practice also requires diagnosing other types of malignancies. Our work addressed this perspective as our dataset comprises different types of thyroid nodules, including papillary, follicular, and Hurthle cell lesions. This diversity in our dataset enhances the robustness and applicability of our model in a real-world clinical setting, making it more comprehensive and effective in diagnosing a broader spectrum of thyroid malignancies.

In several studies, such as the work by Kwon et al. [38], transverse and longitudinal images were collected together for training the model to classify thyroid nodules. In a work conducted by Zhao et al. [39], they proposed a multiscale detection network to detect thyroid nodules and used an attention-based classification network for classifying the detected thyroid nodules. They indicated that their dataset contains transverse and longitudinal views; however, they did not discuss in clear detail how data processing for each view was conducted. Tao et al. [40] extracted features from transverse and longitudinal views and applied feature fusion of the views. They reported that their model with feature fusion achieved better performance compared to separate views. Although we did not use feature fusion and used the ensemble technique instead, our findings were in line with their report on the assessment of transverse and longitudinal views separately. We trained our model with separate views in the current work, and our pipeline achieved a better performance by ensembling models from two views. Gu et al. used an XGBoost pipeline for the assessment of thyroid cancer, and they reported that it has the potential to evaluate thyroid cancers comparable to TIRAD scores [41]. In another study, Wang et al. combined the predictive probability from the ResNet50 and XGBoost models to generate an integrated model to assess thyroid nodules and achieved a diagnostic accuracy of 76.77%, a sensitivity of 69.23%, a specificity of 81.67%. Our deep learning pipeline, which is a combination of YOLOv5 and XGBoost, achieved comparable sensitivity and specificity to the expert radiologist with ten years of experience. Our model did not predict any malignant nodule with ACR-TIRAD > 3 as benign and predicted 88% of the test data with benign biopsy results as benign. In a study, Wei et al. [42] introduced an ensemble of four models for the classification of thyroid nodules, which had a superior performance compared to a radiologist’s evaluation using ACR-TIRAD. It is noted that in certain cases, despite radiologists’ inter-reader agreements, thyroid nodules exhibit complex behaviors, with instances where ultrasound imaging suggests malignant features in ultrasound imaging, yet histopathological examination reveals the nodule is benign. Liu et al. [23] reported that both AI models and radiologists may misclassify these cases to a similar extent. Buda et al. [12] reported a deep learning model with similar sensitivity and specificity to the experts’ consensus based on ACR-TIRAD for thyroid nodule classification and better sensitivity and specificity over 5/9 expert radiologists in clinical practice. In another study, Koh et al. [43] used an ensemble technique to evaluate the deep-learning model with expert radiologists. They also reported that deep learning could have diagnostic performance for differentiating thyroid nodules in internal and external datasets. Current work corroborates these findings, reinforcing their findings and introducing innovative methodologies. Our study proposes a deep learning-based methodology for algorithmically evaluating thyroid nodules. By integrating dual-view analysis and implementing ensemble techniques, our model demonstrates enhanced diagnostic performance. These advancements in AI-driven models present significant potential for improving the accuracy and efficiency of thyroid nodule classification, thereby contributing to optimal clinical practice. Our model development had some shortcomings. Our dataset did not include the nodules with the indeterminate diagnosis from FNA reports, and the patients underwent FNA based on suspicious nodules. We used only B-mode ultrasound modality, and further studies should validate these results on modalities such as color Doppler imaging. We set a threshold for the ACR-TIRADS evaluation and did not include nodule size. In future work, external validation and contributing radiologists to gain inter and intra-reader agreement are considered for the generalizability of our proposed model.

Future work will focus on further validating our approach with larger datasets, conducting ablation studies, and exploring real-time clinical applications.

## 5. Conclusions

In conclusion, our study demonstrates the potential of a deep learning-based pipeline, utilizing transverse and longitudinal views of ultrasound images, for the detection and classification of thyroid nodules. By employing two YOLOv5 models and ensemble their outputs with an XGBoost model, we achieved comparable diagnostic performance to the TIRADS score. Our results highlight the promise of advanced integrated AI techniques in enhancing thyroid nodule evaluation and potentially improving patient outcomes.

## Figures and Tables

**Table 1:** Sensitivity analysis of TIRAD scoring with different thresholds for classifying malignant and benign.

TIRAD Score	Sensitivity	Specificity
TIRAD < 1	97%	11%
TIRAD < 2	97%	13%
TIRAD < 3	78%	34%
TIRAD < 4	36%	86%

**Table 2:** Comparing the model’s performance with the ground truth and ACR-TIRAD score.

Ground Truth	Prediction	TIRAD Score	Percent
Benign	Benign	TIRAD > 3	8.4% (23/81)
Benign	Benign	TIRAD ≤ 3	18.5% (15/81)
Benign	Malignant	TIRAD ≤ 3	0
Benign	Malignant	TIRAD > 3	6.17% (5/81)
Malignant	Malignant	TIRAD > 3	33.3% (27/81)
Malignant	Malignant	TIRAD ≤ 3	3.7% (3/81)
Malignant	Benign	TIRAD > 3	6.17% (5/81)
Malignant	Benign	TIRAD ≤ 3	3.7% (3/81)

## Data Availability

Data generated or analyzed during the study are available from the corresponding author by reasonable request.
